# Changes in profile of lipids and adipokines in patients with newly diagnosed hypothyroidism and hyperthyroidism

**DOI:** 10.1038/srep26174

**Published:** 2016-05-19

**Authors:** Yanyan Chen, Xiafang Wu, Ruirui Wu, Xiance Sun, Boyi Yang, Yi Wang, Yuanyuan Xu

**Affiliations:** 1 Department of Geriatrics, the First Affiliated Hospital, China Medical University, Shenyang, Liaoning, P. R. China; 2 Program of Environmental Toxicology, School of Public Health, China Medical University, Shenyang, Liaoning, P. R. China; 3 Department of Occupational and Environmental Health, College of Public Health, Dalian Medical University, Dalian, Liaoning, P. R. China; 4 Environment and Non-communicable Disease Research Center, School of Public Health, China Medical University, Shenyang, Liaoning, P. R. China

## Abstract

Changes in profile of lipids and adipokines have been reported in patients with thyroid dysfunction. But the evidence is controversial. The present study aimed to explore the relationships between thyroid function and the profile of lipids and adipokines. A cross-sectional study was conducted in 197 newly diagnosed hypothyroid patients, 230 newly diagnosed hyperthyroid patients and 355 control subjects. Hypothyroid patients presented with significantly higher serum levels of total cholesterol, triglycerides, low-density lipoprotein cholesterol (LDLC), fasting insulin, resistin and leptin than control (*p* < 0.05). Hyperthyroid patients presented with significantly lower serum levels of high-density lipoprotein cholesterol, LDLC and leptin, as well as higher levels of fasting insulin, resistin, adiponectin and homeostasis model insulin resistance index (HOMA-IR) than control (*p* < 0.05). Nonlinear regression and multivariable linear regression models all showed significant associations of resistin or adiponectin with free thyroxine and association of leptin with thyroid-stimulating hormone (*p* < 0.001). Furthermore, significant correlation between resistin and HOMA-IR was observed in the patients (*p* < 0.001). Thus, thyroid dysfunction affects the profile of lipids and adipokines. Resistin may serve as a link between thyroid dysfunction and insulin resistance.

Hypothyroidism and hyperthyroidism are two of the most common thyroid diseases. Both are characterized by abnormal circulating levels of thyroid hormones and thyroid-stimulating hormone (TSH). Thyroid hormones serve as regulators of various processes in the body. They stimulate resting metabolic rate and heat production, influence cell proliferation and development, modulate response to other hormones, and change metabolism of carbohydrate, protein and lipids^1^,^2^,^3^. Thyroid dysfunction results in alterations of appetite, body weight, muscle mass and adipose tissue. In addition to typical clinical symptoms directly related to thyroid hormones and TSH, patients with thyroid dysfunction are likely to present with high incidences of insulin resistance, type 2 diabetes and cardiovascular diseases^4^,^5^,^6^,^7^. However, the underlying mechanisms are not fully understood.

Adipose tissue consists of multiple types of cells, such as adipocytes, immune cells, and fibroblasts. It is classified as white adipose tissue and brown adipose tissue, and the former is predominant after birth. The discovery of the obese gene, leptin, led people to realize that white adipose tissue is a key endocrine organ in addition to its effect on energy reservation^8^. White adipose tissue secrets a variety of hormones, cytokines and growth factors, which have effects on both local tissue and distant organs and tissues throughout the body. Adiponectin, resistin and leptin are of these adipocyte-derived hormones, namely adipokines. They are involved in many metabolic disorders, including obesity and type 2 diabetes, as well as hypertension and cardiovascular diseases, by promoting inflammation of adipocytes^9^. These adipokines and thyroid hormones share some physiological effects such as regulating energy expenditure and metabolism of glucose and lipids^10^,^11^. So it is conceivable that there is interaction between the thyroid axis and action of adipose tissue. Thyroid dysfunction may influence action of adipose tissue, which contributes to other metabolic disorders. In line with this, changes in lipolysis also occur in patients with thyroid dysfunction^12^,^13^. Though some reports are controversial, in most cases serum levels of lipids are found to increase in hypothyroidism but to decrease in hyperthyroidism^14^. In addition, adipocytes express high levels of TSH receptors which function similar to those in thyroid^15^, indicating that TSH participates in the regulation of adipocyte functions including secreting adipokines.

Studies investigating thyroid disorders and their consequences on adipokine profiles are limited and results are highly variable and conflicting. Therefore, in the present study, we evaluated serum levels of adiponectin, resistin and leptin, as well as cholesterol and triglycerides in patients with newly diagnosed patients with hypothyroidism and hyperthyroidism. Furthermore, associations of circulating levels of lipids or adipokines with thyroid hormones, TSH or insulin resistance were also investigated.

## Results

### Demographic and clinical characteristics of study subjects

Characteristics of study subjects are shown in Table 1. Of 782 study participants, there were no significant differences in average age or gender ratio between control, hypothyroid and hyperthyroid groups. As expected, levels of serum free thyroxine (FT4) and free tri-iodothyronine (FT3) were lower in hypothyroid group but higher in hyperthyroid group compared to those in control (*p* < 0.05). Levels of TSH were significantly increased in hypothyroid but decreased in hyperthyroid group compared to control (*p* < 0.05). Body mass index (BMI) was highest in hypothyroid group but lowest in hyperthyroid group (*p* < 0.05, compared to control). Though serum fasting glucose levels were not significantly different between each group, a significant increase in fasting insulin levels of hypothyroid and hyperthyroid patients was observed (*p* < 0.05). Homeostasis model insulin resistance index (HOMA-IR) was significantly elevated in hyperthyroid group compared to control but not in hypothyroid group.

### Serum levels of lipids and adipokines

**Figure 1:**
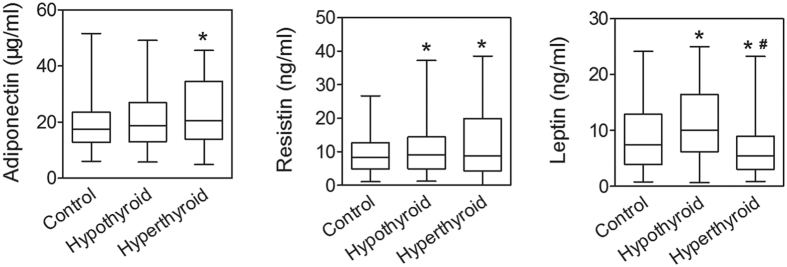
Serum levels of adiponectin, resistin and leptin in control, hypothyroid and hyperthyroid groups. Data are expressed as box and whiskers (medium and min to max). *p < 0.05 compared with control. ^#^*p* < 0.05 compared with hypothyroid group.

Changes in serum profile of lipids and adipokines can reflect altered function of adipocytes. We first examined lipid profile (Table 1). Patients with hypothyroidism presented with significantly higher serum levels of total cholesterol (TC), triglycerides (TG) and low-density lipoprotein cholesterol (LDLC) than control (*p* < 0.05) (Table 1). Patients with hyperthyroidism presented with significantly lower serum levels of high-density lipoprotein cholesterol (HDLC) and LDLC than control (*p* < 0.05). The alteration of the serum lipid profile is consistent with previous reports^13^,^14^,^16^,^17^. Results of adipokines were shown in Fig. 1. Hyperthyroid group exhibited significantly higher serum levels of adiponectin compared to control (*p* < 0.05). There was no significant difference in levels of serum adiponectin between hypothyroid and control groups. Both hypothyroid and hyperthyroid groups exhibited higher serum levels of resistin compared to control (*p* < 0.05). Serum levels of leptin were elevated in hypothyroid group but decreased in hyperthyroid group (*p* < 0.05).

### Associations of adipokines or HOMA-IR with thyroid hormones or TSH

**Figure 2:**
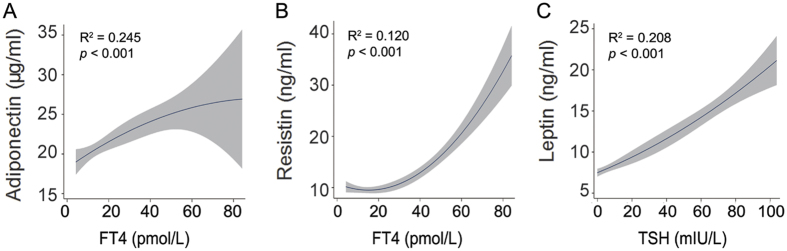
Associations of serum adipokines with FT4 or TSH analyzed by quadratic regression model. Plots show the association between serum adiponectin, resistin or leptin and FT4 or TSH as predicted mean with 95% confidence interval.

The outcomes of non-linear regression models investigating the association between adiponectin, resistin, leptin, or HOMA-IR and FT4, FT3 or TSH are shown in Supplemental Table 1. Of 12 statistical tests, FT4 levels best explained the variability in resistin and adiponectin levels (12% and 24.5%, respectively, Fig. 2A,B), whereas TSH levels best explained the variability in leptin levels (20.8%, Fig. 2C). Only a week contribution of FT4, FT3 or TSH (0.2%–1.4%) to variability in HOMA-IR was observed (Supplemental Table 1). Moreover, the relationships between resistin or adipokines and FT4 exhibited a (reverse) U-shape, indicating correlation between theses adipokines and FT4 varies in different thyroid states. Thus, association analyses of adipokines of interest or HOMA-IR with FT4, FT3 or TSH were also conducted using multivariable linear regression models in control, hypothyroid and hyperthyroid groups separately (Table 2). Serum levels of adiponectin were positively associated with FT4 in control and hypothyroid groups, but not in hyperthyroid group. Serum levels of resistin were positively associated with FT4 in control and hyperthyroid groups, whereas negatively associated with FT4 in hypothyroid group. In the hyperthyroid group, serum levels of resistin also showed positive association with FT3 but negative association with TSH. Serum levels of leptin were positively associated with TSH in all the three groups, and negatively associated with FT3 in control and hyperthyroid groups. Results of the non-linear analyses in a combined group and multivariable linear regression analyses in separate groups are consistent, indicating that there are significant associations between adipokines and thyroid function. With respect to HOMA-IR, only positive association with FT3 in hypothyroid group in multivariable linear regressions was observed. In addition, we did not find significant associations of TC, TG, HDLC and LDLC with thyroid hormones or TSH.

### Correlations between HOMA-IR and adipokines

**Figure 3:**
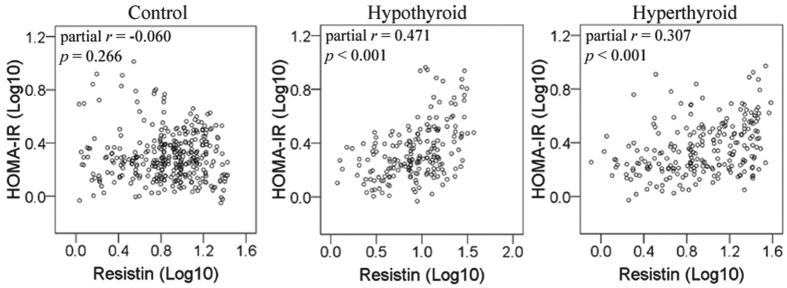
Correlation between HOMA-IR and serum levels of resistin in control, hypothyroid and hyperthyroid groups. Partial *r* was assessed by linear regression model with age, gender, BMI, TC, TG, HDLC and LDLC as independent variables.

Potential correlations between HOMA-IR and serum levels of adiponectin, resistin and leptin were investigated in seperate groups. HOMA-IR was positively associated with serum levels of resistin in hypothyroid group (partial *r* = 0.471, *p* < 0.001) and in hyperthyroid group (partial *r* = 0.307, *p* < 0.001) but not in control (Fig. 3). No significant correlations were observed between HOMA-IR and serum levels of adiponectin or leptin.

## Discussion

Dyslipedemia has been reported in patients with thyroid dysfunction^13^,^14^,^16^,^17^. In the present study, patients with hypothyroidism showed significant increase in serum levels of TG, TC and LDLC, which is compatible with previous reports. Decreased levels of thyroid hormones attenuate activity of lipoprotein lipase (LPL), the enzyme responsible for clearance of TG-rich lipoproteins^18^,^19^, and thus lead to increased levels of TG in the serum. Thyroid hormones such as T3 have been demonstrated to regulate LDL receptors by directly binding to thyroid hormone responsive elements (TREs)^20^ and controlling sterol regulatory element-binding protein^21^. In hypothyroidism, decreased thyroid hormones lead to reduced expression of LDL receptors, which may attenuate cellular uptake of LDLC from circulation and catabolism of LDLC and finally result in increased levels of circulating TC^14^,^22^. The ratio of LDLC to HDLC is considered as a prognostic marker for cardiovascular disease. Elevated ratio of LDLC to HDLC occurred in patients with hypothyroidism due to a significant increase in LDLC levels and a slight decrease in HDLC levels, indicating increased risk of cardiovascular disease. However, in patients with hyperthyroidism, serum levels of HDLC and LDLC decreased significantly and serum levels of TC had a tendency to decrease. As mentioned previously, thyroid hormones are able to regulate expression of LDL receptor, which leads to altered cellular uptake and catabolism of LDL particles^14^,^22^,^23^. LPL activity stimulated by increased levels of thyroid hormones may also contribute to decreased circulating levels of lipoproteins^18^. In addition, thyroid hormones modulate HDLC metabolism by increasing activity of cholesteryl ester transfer protein, which exchanges high density lipoproteins to very low density lipoproteins^24^.

Insulin resistance is a state of glucose homeostasis in which insulin produces a less-than-expected biological effect at the liver, muscle, adipose tissue and other body tissues. A deficiency or an excess of thyroid hormones has been demonstrated to induce development of insulin resistance and disrupt glucose metabolism^5^,^6^,^25^,^26^. We observed a significant rise in HOMA-IR in hyperthyroid group and a slight increase in HOMA-IR in hypothyroid group. These data correspond with previous studies indicating that insulin resistance was associated with hypothyroidism and hyperthyroidism^4^,^5^,^6^. Insulin resistance is classified into peripheral and hepatic types. In hypothyroidism, peripheral insulin resistance developed in skeletal muscle and adipose tissue is suggested, whereas in hyperthyroidism, both hepatic and peripheral insulin resistance is observed^6^,^26^,^27^. Other etiological mechanisms of insulin resistance in thyroid dysfunction include altered expression of glucose transporters on monocytes^28^,^29^ and changed blood flow in peripheral tissues^30^.

Adiponectin is mainly derived from adipose tissue and circulates at a high concentration in human plasma. It acts as an insulin sensitizer and is involved in anti-inflammatory and anti-atherogenic effect^31^,^32^,^33^,^34^,^35^. Individuals with obesity, insulin resistance and diabetes are usually presented with decreased circulating levels of adiponectin^32^,^33^,^34^. In previous epidemiological and experimental studies, conflicting results of association between thyroid hormones and adiponectin levels were reported^36^. No definitive conclusions can be drawn. Here, we observed positive associations between serum levels of adiponectin and FT4 in control and patients with hypothyroidism, but not in patients with hyperthyroidism in linear regression, which is consistent with the curve estimation in a combined group. It is noteworthy, however, that levels of adiponectin were significantly increased in patients with hyperthyroidism. The reason for this adiponectin elevation is unclear. Insulin resistance of hepatic and peripheral tissues occurs in hyperthyroidism. Given that adiponectin has anti-diabetic property and negative correlation with insulin resistance^32^,^33^,^34^, the increase in adiponectin levels might be a compensatory mechanism against insulin resistance in hyperthyroidism. However, we did not find significant association between serum levels of adiponectin and HOMA-IR. This might be due to differences in patients’ characteristics, including duration and degree of thyroid dysfunction, metabolic effects of other hormones, and possible effects of intermediate metabolism.

Resistin is a cysteine-rich polypeptide, which antagonizes insulin effect and causes insulin resistance. It is secreted by both adipose tissue^37^,^38^ and adipose tissue-infiltrated macrophages at the site of inflammation^39^. Information on the association between thyroid functions and resistin is limited and not consistent^37^,^40^,^41^,^42^,^43^. In our study, higher serum levels of resistin in patients with hypothyroidism and hyperthyroidism were observed. This is consistent with Koyuncu *et al*., which was conducted in Turkey with 15 cases of both hypothyroidism and hyperthyroidism^44^. The relationship between resistin and FT4 exhibited a U-shape in nonlinear regression, indicating varied mechanisms are involved in the relationship between thyroid hormones and resistin in different thyroid states. Moreover, there was significant correlation between HOMA-IR and resistin in patients with thyroid dysfunction. Increased resistin may lead to a chronic subinflammatory state that plays a central role in the development of insulin resistance, type 2 diabetes and cardiovascular diseases^45^. Thus, our data suggest that resistin may be a link between thyroid dysfunction and insulin resistance and at least serve as a biomarker for insulin resistance and probably other diseases.

Leptin is considered to improve peripheral insulin sensitivity and modulate pancreatic β-cell function in addition to regulate energy consumption and body weight^46^. It is predominantly produced by adipocytes. Serum levels of leptin are proportional to BMI. In the present study, BMI was significantly different between control and subjects with thyroid dysfunction. So it’s not a surprise to see reduced leptin levels in patients with hyperthyroidism (low BMI), but elevated in patients with hypothyroidism (high BMI). In addition, association between serum leptin and thyroid function may be partly mediated by TSH. TSH has a direct effect on leptin secretion by simulating TSH-receptor on adipocytes^35^. Recent studies provide evidence for a link between serum levels of TSH and leptin in young men with euthyroid state and in obese individuals^47^,^48^, respectively. There are many studies investigating circulating leptin levels in thyroid disorders. However, the results are highly controversial. Our data are consistent with those from Guzel *et al*., in which serum levels of leptin were significantly higher in overt and subclinical hypothyroid patients^49^. Some studies found lack of change in leptin levels with thyroid dysfunction^50^,^51^,^52^,^53^. For instance, thyroid dysfunction was of recent onset and patients usually presented lack of change in body mass. Many Chinese residents don’t get regular physical examinations. Their health problems often go undiscovered until they go to clinics with obvious clinical symptoms. The newly diagnosed patients in the present study may have previous thyroid dysfunctions and thus showed significant change in BMI and adipokine profile.

Our study has some limitations. First, this was a cross-sectional study, which does not allow us to make causal inferences between variables, such as adipokines, thyroid hormones and insulin resistance. A bidirectional interaction between adipokines and hypothalamus–pituitary–thyroid axis is suggested by previous study^28^. The significant associations between adipokines and thyroid hormones or TSH, in particular in euthyroid subjects, in the present study support this idea as well. Secondly, though the age and gender were not significantly different between study groups, there was still difference in distribution among groups. Also, there are many causes for hyperthyroidism. We did not classify the cases into different subgroups according to the causes, which would result in small sample sizes of subgroups. Moreover, although mean levels of leptin, adiponectin and resistin were different between patients with hypothyroidism and/or hyperthyroidism and euthyroid controls, there is a large overlap between the groups, suggesting that in addition to thyroid state other key factors are involved in the regulation of these adipokines.

## Methods

### Study subjects

A total of 427 cases (197 hypothyroid patients and 230 hyperthyroid patients) and 355 control subjects with normal thyroid function were conducted at the First Affiliated Hospital of China Medical University, Shenyang, China from July 2014 to December 2014. Inclusion criteria were age of 18 to 75, newly diagnosed and untreated patients with thyroid dysfunction. Exclusion criteria included a history of thyroid disease, diabetes, hypertension, stroke, liver disease and any other known diseases, pregnancy within the last two years, and any medications that could change thyroid hormone levels or lipid metabolism. The study protocol was approved by the ethics committee at China Medical University. The methods were carried out in accordance with the approved guidelines. All subjects gave written informed consent. Hyperthyroid patients were diagnosed with elevated serum levels of FT3 and/or FT4 but decreased TSH levels compared to reference ranges (FT4, 9.01–19.05 pmol/L; FT3, 2.63–5.70 pmol/L; and TSH, 0.35–4.94 mIU/L). Hypothyroid patients were diagnosed with decreased serum levels of FT3 and/or FT4 but elevated serum levels of TSH.

### Sample analysis

After overnight fasting, peripheral venous blood was collected from all study subjects. Serum was isolated according to standard protocol. Serum levels of FT3 and FT4 and TSH were measured by solid-phase chemiluminescence immunoassay (IMMULITE1000, DPC, USA). Serum concentrations of TC, TG, HDLC and LDLC were measured using an automatic biochemical analyzer (AU1000, Olympus, Japan). Concentrations of glucose and insulin were measured by Hexokinase end point method and chemiluminescent analysis, respectively. Insulin resistance was estimated with HOMA-IR, which was calculated as serum glucose concentration (mmol/L) x serum insulin (FINS) concentration (mIU/L)/22.5. Serum concentrations of adiponectin, resistin and leptin were measured by quantitative sandwich enzyme immunoassays (CUSABIO, Wuhan, China). BMI was calculated as weight in kilograms divided by height in meters squared (kg/m^2^).

### Statistical analyses

Statistical analyses were conducted by using SPSS software (version 13.0, SPSS, Chicago, IL, USA) or Stata software (Version 12.1, StataCorp LP, College Station, TX, USA). Age- and gender-adjusted comparisons between control, hypothyroid and hyperthyroid subjects were conducted by using one-way analysis of variance (ANOVA) test and Bonferroni test for post-hoc multiple comparisons. Data of normal distribution were expressed as mean ± SD. Data not meeting with normal distribution were log transformed before further analyses and expressed as media and range. Multivariable linear regression analyses were then applied to evaluate associations of HOMA-IR, serum lipid parameters or adipokines with FT4, FT3 or TSH, as well as associations of HOMA-IR with serum lipid parameters or adipokines. Nonlinearity of the association between serum adipokines and FT4, FT3 or TSH levels was analyzed by quadratic regression model. Statistical significance was considered achieved at a value of *p* < 0.05.

## Additional Information

**How to cite this article**: Chen, Y. *et al*. Changes in profile of lipids and adipokines in patients with newly diagnosed hypothyroidism and hyperthyroidism. *Sci. Rep.* **6**, 26174; doi: 10.1038/srep26174 (2016).

## Supplementary Material

Supplementary Information

## Figures and Tables

**Table 1:** Characteristics of control, hypothyroid and hyperthyroid subjects.

	Control	Hypothyroid	Hyperthyroid
n	355	197	230
Male	120 (33.8%)	68 (34.5%)	76 (33.0%)
Age (year)	47.07 ± 15.15	46.50 ± 15.64	45.85 ± 13.94
FT4 (pmol/L)	14.29 (10.00, 19.04)^a^	7.25 (4.28, 9.68)^b^	32.18 (5.21, 84.09)^c^
FT3 (pmol/L)	3.38 (2.63, 6.65)^a^	2.57 (1.08, 4.67)^b^	15.97 (1.81, 59.63)^c^
TSH (mIU/L)	2.52 (0.51, 4.94)^a^	3.58 (4.98, 103.59) ^b^	0.004 (0.000, 16.43)^c^
BMI	24.55 ± 3.36^a^	25.72 ± 2.89^b^	23.76 ± 2.91^c^
Glucose (mmol/L)	4.44 (2.38, 16.51)	4.57 (2.23, 13.79)	4.52 (2.09, 9.92)
Insulin (mU/L)	9.07 (3.64, 27.90)^a^	9.74 (3.95, 29.35)^b^	10.15 (5.89, 30.74)^b^
HOMA-IR	1.89 (0.89, 10.29)^a^	1.95 (0.93, 9.19)^ab^	2.12 (0.94, 9.36)^b^
TC (mmol/L)	4.65 ± 0.89^a^	4.80 ± 0.85^b^	4.55 ± 0.87^a^
TG (mmol/L)	1.53 ± 1.30^a^	1.80 ± 1.18^b^	1.50 ± 1.25^a^
HDLC (mmol/L)	1.30 ± 0.30^a^	1.24 ± 0.44^ab^	1.22 ± 0.30^b^
LDLC (mmol/L)	2.50 ± 0.89^a^	2.68 ± 1.12^b^	2.32 ± 0.95^c^

Comparisons of variables expect for age and gender between groups were adjusted by age and gender. Different superscripts indicate significantly statistical difference between groups (*p* < 0.05).

**Table 2:** Multivariable regression analyses of associations of adipokines and HOMA-IR with thyroid hormones and TSH.

	FT4		FT3		TSH	
	*β* (95% CI)	*p*	*β* (95% CI)	*p*	*β* (95% CI)	*p*
Control						
Adiponectin	0. 746 (0.489, 1.004)	<0.001	−0.014 (−0.238, 0.209)	0.899	−0.026 (−0.108, 0.056)	0.540
Resistin	1.320 (0.861,1.780)	<0.001	0.060 (−0.338, 0.459)	0.766	−0.018 (−0.164, 0.129)	0.813
Leptin	−0.337 (−0.882 0.208)	0.225	−0.663 (−1.111, −0.215)	0.004	1.134 (1.018, 1.250)	<0.001
HOMA-IR	−0.009 (−0.279, 0.260)	0.947	0.047 (−0.177, 0.270)	0.682	−0.036 (−0.118, 0.046)	0.386
Hypothyroid						
Adiponectin	0.732 (0.399, 1.065)	<0.001	−0.009 (−0.243, 0.225)	0.940	0.060 (−0.007, 0.128)	0.080
Resistin	−1.064 (−1.586, −0.542)	<0.001	0.239 (−0.125, 0.602)	0.197	0.004 (−0.103, 0.110)	0.948
Leptin	−0.555 (−1.165, 0.056)	0.075	0.025 (−0.381, 0.432)	0.902	0.631 (0.557, 0.705)	<0.001
HOMA-IR	0.104 (−0.277, 0.485)	0. 590	0.352 (0.104, 0.600)	0.006	0.005 (−0.067, 0.078)	0.882
Hyperthyroid						
Adiponectin	0.148 (−0.006, 0.303)	0.060	0.117 (0.020, 0.214)	0.018	−0.009 (−0.036, 0.018)	0.502
Resistin	0.903 (0.679, 1.128)	<0.001	0.408 (0.257, 0.558)	<0.001	−0.081 (−0.123, −0.038)	<0.001
Leptin	−0.236 (0.459, −0.014)	0.038	−0.161 (−0.300, −0.021)	0.025	0.172 (0.204, 0.602)	<0.001
HOMA-IR	−0.036 (−0.165, 0.094)	0.588	0.027 (−0.054, 0.109)	0.511	−0.011 (−0.033, 0.011)	0.326

Multivariable regression analyses were conducted with age, gender, BMI, TC, TG, HDLC and LDLC as independent variables. Analyses were conducted in control, hypothyroid and hyperthyroid group separately.
