# Supplementary material for: Changes in profile of lipids and adipokines in patients with newly diagnosed hypothyroidism and hyperthyroidism
Source: Sci Rep. 2016 May 19;6:26174. doi: 10.1038/srep26174 (PMC4872157; doi:10.1038/srep26174)
Supplement: Supplementary Information [file srep26174-s1.pdf]

Changes in profile of lipids and adipokines in patients with newly diagnosed hypothyroidism and hyperthyroidism

Yanyan Chen [2], Xiafang Wu [1], Ruirui Wu [1], Xiance Sun [3], Boyi Yang [4], Yi Wang [4*] and Yuanyuan Xu [1*]

[1] Program of Environmental Toxicology, School of Public Health, China Medical University, Shenyang, Liaoning, P. R. China..

[2] Department of Geriatrics, the First Affiliated Hospital, China Medical University, Shenyang, Liaoning, P. R. China.

[3] Department of Occupational and Environmental Health, College of Public Health, Dalian Medical University, Dalian, Liaoning, P. R. China.

[4] Environment and Non-communicable Disease Research Center, School of Public Health, China Medical University, Shenyang, Liaoning, P. R. China.

Corresponding to Yuanyuan Xu and co-corresponding to Yi Wang

Address: No.77 Puhe Road, Shenyang North New Area, Shenyang, Liaoning Province, 110122, P. R. China.

* Correspondence and request for materials should be addressed to Y.X. (email: yyxu@mail.cmu.edu.cn) or to Y.W. (email: wangyi@mail.cmu.edu.cn).

Supplementary Information

Supplemental Table 1. The results of quadratic regression model between adipokines or HOMA-IR and FT4, FT3 or TSH in serum.

	FT4		FT3		TSH	
	R^2	*p*	R^2	*p*	R^2	*p*
Resistin	0.120	< 0.001	0.058	<0.001	<0.001	0.915
Leptin	0.092	< 0.001	0.055	<0.001	0.208	<0.001
Adiponectin	0.245	< 0.001	0.023	<0.001	0.004	0.240
HOMA-IR	0.002	0.510	0.008	0.042	0.014	0.004
